# The Anatomy, Physiology, Pathology, and Remedial Treatment of the Fifth Pair of Nerves

**Published:** 1860-11

**Authors:** J. H. M‘Quillen


### THE ANATOMY, PHYSIOLOGY, PATHOLOGY, AND REMEDIAL TREATMENT OF THE FIFTH PAIR OF NERVES.

BY J. H. M'QUILLEN, D. D. S.

(Continued.)

The marked resemblance between the spinal nerves and the fifth pair of nerves in their mode of origin by two roots, anterior and posterior, the ganglionic expansion upon the latter, the union of the anterior root with the inferior maxillary nerve, the division, subdivision, distribution and connection of the different branches with each other and with other nerves, and with the cranial sympathetic ganglion, having been described and illustrated, the next step is to consider the functions of this remarkable nerve.

To determine these functions, the most extended investigations and experiments have been made by numerous observers, among whom Sir Charles Bell, Magendie, Mayo, and Bernard have been the most indefatigable and successful. Although physiologists are united in opinion, except in some collateral details, there still appears to be some important points which can not be regarded as definitely settled. Deferring the consideration of these points until they come up in proper order, it may be said in the beginning that the analogy between the larger or gaglionic root of the fifth pair and the posterior spinal root, and between the smaller root of the fifth and the anterior spinal root, affords a clue by which, with the present knowledge of the functions of the roots of the spinal nerves, it would be natural to infer that the larger consists of sensitive, and the smaller of motor fibres. The correctness of this inference is confirmed by the distribution of the different branches, by pathological conditions of the nerve in human beings, and by experiments upon the lower animals.

Thus, on tracing each of the three great divisions of the nerve, the ophthalmic and superior maxillary branches, which are composed of fibres derived exclusively from the larger root, are found to be distributed only to sentient surfaces, viz: the integuments of the forehead, temples, eyelids, greater part of the ear, conjunctiva, Schneiderian membrane, mucous membrane of the mouth and upper part of the pharynx, and the pulps of the upper teeth, and are unquestionably the sensitive nerves of those regions. The *inferior* maxillary branch, in contradistinction to the ophthalmic and superior maxillary, by its union with the smaller root, has two distinct sets of branches, one by which the muscles of mastication, viz: the temporal, masseter, buccal and two pterygoid, derive their motor impulse; and the other, distributed to the pulps of the lower teeth, integuments of the lower lip and chin, and the mucous membrane of the mouth and tongue, and supplying those parts with common sensibility. In addition, according to the generally received opinion among physiologists, the lingual branch is the nerve of taste for the anterior part of the tongue.

Of the pathological conditions illustrative of function, Sir Charles Bell cites a number of cases, in one of which a small sacculated tumor affected the roots of this nerve, so that the sensibility was destroyed in the parts to which its branches are distributed. In another case, in an effort to extract a tooth from the lower jaw of a gentleman, the inferior dental nerve was so much injured, that the half of the lip to which the nerve is distributed after emerging from the jaw was rendered completely insensible, and when he put a tumbler to his mouth, it felt as if a piece had been broken out. Precisely the same thing occurred in another case, from division of the infra-orbital branch of the fifth pair, which sends filaments to one-half of the upper lip. "A gentleman falling, a sharp point entered his cheek, and divided the infra-orbital nerve; the effect was loss of sensation, without loss of motion, in that half of the upper lip to which the nerve is distributed. The remarkable circumstance was, that this individual made the remark, when a cup was put to his lip, that they had given him a broken one. The part of the cup which was placed in contact with the insensible portion of the lip appeared to him to be broken off."

As the result of experimental observation upon the inferior animals, it is found, on irritating the ophthalmic and superior maxillary nerve, that a sensation of acute pain is induced in the parts to which they are distributed, but no convulsive movements occur. By division of the branches the sensibility of the parts is completely destroyed. On the other hand, if the electro-galvanic current is applied to the smaller or motor root of the fifth pair of an animal just dead, the most powerful action is excited in the jaws, which are snapped and ground together with considerable force.

This experiment, which is a very interesting and instructive one to witness, is quite easy to perform. Thus, immediately after an animal is killed, the cranium should be opened, and then carefully removing the brain, the ganglion of Gasser will be readily found; on gently raising this, the motor root of the fifth pair will be observed beneath it. Applying the electric current to this, the results already described will be readily obtained. The animals most easily procured for purposes of experiment are rabbits, cats, and dogs. Excellent and unobjectionable opportunities for prosecuting such investigations could no doubt be afforded in the slaughter-houses of beef and mutton butchers.

In addition to the satisfactory response obtained in the above experiment, which is not open to the objection of torturing the brute creation, a more objectionable operation to many, upon a living animal, affords the most decided and conclusive evidence of the function of this branch. Thus, when the inferior maxillary nerve is divided on both sides in a living animal, the lower jaw falls, from paralysis of the masticatory muscles. When the section is confined to one side, the parallelism of the jaw is lost, even in a state of repose, but particularly in the act of chewing. In addition to this loss of motion—which, by-the-by, is confined to the muscles of mastication, and does not include the superficial muscles of expression, as they are under the control of the portio dura —the sensibility of the lower part of the face and tongue and the sense of taste throughout the anterior two-thirds of the tongue are destroyed.

The fifth nerve may therefore be regarded as the sensitive nerve to that great surface, both internal and external, which comprises the anterior and antero-lateral parts of the face and head, and as the motor nerve of mastication. In addition to this, according to recent investigations of Bernard, a motor branch is given off to the parotid glands, which possesses exclusively the power of stimulating their action. This branch is from the auriculo-temporal nerve, which is derived from the inferior maxillary.

The branches of the greater or ganglionic portion of the fifth pair, although formed of sensitive filaments exclusively, exercise a decided influence on the movements of the muscles of the head and face, and other parts to which they are distributed, through the numerous anastomoses which take place between these branches and the facial and hypoglossal nerves and the nerves of the muscles of the eye. This is due to the fifth pair providing the muscles with that sensibility, without which the mind, being unconscious of their position and state, can not voluntarily exercise them. Thus it may be an excitor to the facial, as in winking; or to the respiratory nerves, as when cold water is dashed in the face; or in sneezing, an act which is frequently induced in some persons by the impression made upon the branches of the fifth pair distributed to the eye, on passing suddenly into a bright light. All of these are instances of purely reflex action.

This naturally leads to the point, that one of the most important and peculiar features connected with the fifth pair is its influence over the organs of special sense. This appears to be owing to some connection between this nerve and the function of nutrition. For it has been frequently observed that in a brief time after complete paralysis, or division of this nerve, the power of all the organs of the special senses may be lost. They do not lose merely their sensibility to common impressions, for which they all depend directly on the fifth nerve, but also their sensibility to the several peculiar impressions for the reception and conduction of which they are purposely constructed and supplied with special nerves. The facts observed in these cases can only be accounted for by the supposition that the function of nutrition in these organs is under the influence of the fifth pair, and that when that nerve is injured, nutrition becomes deranged, and consequently interferes with the due performance of the special functions of the different organs.

Thus Magendie and Longet found that division of the fifth pair within the cranium of rabbits produced a decided effect on the nutrition of the corresponding eye, with, however, this remarkable difference: if, for instance, the division was made posterior to the ganglion of Gasser, partial opacity of the cornea alone followed; but if the ophthalmic branch was divided, anterior to the ganglion, a low, destructive inflammation ensued, within a period varying from twenty-four hours to a week, in the conjunctiva, sclerotica, and interior parts of the eye, and which, in the majority of cases, progressed until ulceration of the cornea and discharge of the different humors of the organ eventually supervened.

The great difference, resulting from these two experiments has led physiologists to infer that a conjoint influence of the sympathetic and the fifth pair is exercised in the nutrition of the special organs of sense; and although the correctness of this position has not as yet been satisfactorily determined, the connection of the ophthalmic, spheno-palatine, otic, and submaxillary ganglia, with the three great divisions of the fifth pair, and the fact that each of the ganglia sends off branches which are distributed to the different organs, give more than an air of mere plausibility to it.

The intimate connection between the sense of sight and the fifth pair is shown in frequent cases where total blindness has followed injury of the frontal nerve as it emerges from the supra-orbital foramen; although in some the blindness ensues immediately as if from concussion of the retina, in others it comes on so gradually and with a low inflammatory disorganization, followed by atrophy of the whole eye, that it appears to be due to defective nutrition.

As already stated, the fifth pair exerts a controlling influence over all the organs of special sense, and it is not alone the sense of vision that is impaired or destroyed in injury or division of the fifth nerve, but the sense of smell, of hearing, and of taste may be affected by the same cause.

Thus the olfactory nerve and the nasal branch of the fifth pair are distributed, for example, to the Schneiderian membrane of the nasal passages; the first, according to physiologists, to endow it with the special sense of smell; the second to supply it with common sensation. It is asserted that after destruction of the olfactory in the lower animals, the power of distinguishing odors, properly so called, is lost, but the Schneiderian membrane remains susceptible to the action of acrid vapors, such as ammonia, horse-radish, mustard, etc. These, however, merely act as irritating substances, affecting the mucous membrane in the same way that they would any other part endowed with general sensibility. After division of the fifth pair, not only is the general sensibility destroyed, but the power of smell is also lost.

In opposition to the above almost universally received opinion among physiologists, Magendie inferred from experiments upon dogs, that he had proved that smell continued after the olfactory nerves had been destroyed. He found, for instance, that a dog which survived division of the olfactory for a considerable period, when food was offered to it rolled up in paper, would unroll the paper and expose and eat the food, taking no notice at the same time of other packages containing blocks of wood. These experiments have been rejected as valueless; but within the past few years Claude Bernard has cited a case where there was a congenital deficiency of the olfactory nerve, which, if the statements of persons from whom he obtained his information are to be relied upon, goes far to strengthen Magendie's opinion that the fifth pair, rather than the olfactory nerve, is the seat of the function of smell.

The case referred to, which may be found in Bernard's works, and in Lewis' Physiology of Common Life, though, strange to say, it has been entirely overlooked by the systematic writers on physiology, came under Bernard's notice when he was Magendie's assistant at the College of France. A woman who had died of consumption was selected by him as a subject for dissection; on opening the skull and removing the brain, he was surprised at not finding the slightest trace of the olfactory nerve. That this was not a case of absence from disease, but of congenital malformation, was proved by the fact that in all other respects the brain was of the normal structure, the origin and distribution of the different nerves being perfect and regular, with the exception of the olfactory, of which neither the bulb nor nerve was present. Two engravings, representing the brain and the base of the skull—which are still preserved in the College of France—are presented in Bernard's works.

The singular phenomena presented in this case excited Bernard's curiosity, and without giving any clue as to the object of his inquiry, he visited different persons with whom the subject (Marie) had been on terms of close intimacy during her life, and found, in response to his inquiries, "that the odor of tobacco proved insupportable to her, and that, on entering a room in the morning where any one had been smoking over night, her first act was to open the window to let out the smell of the stale tobacco; that she frequently complained of the fetid smell of a closet which was near her room; that she was fond of flowers, and always smelled of them;" and, in fact, that her likes and dislikes, with regard to pleasant and disagreeable odors, were as decided as those manifested by persons generally. From this testimony, which Bernard regards as reliable, it is quite evident that the sense of smell was possessed by the individual, and "as the olfactory nerve was absent and the other nerves were in a perfect condition, it appears to be strong corroborative evidence that the filaments of the fifth pair distributed to the Schneiderian membrane served the function of smell, as those which are furnished to the tongue serve the function of taste."

As Bernard suggests, the most reliable experiment that can be performed to determine the relative power of the olfactory and the fifth pair, in the sense of smell, would be to select a well-trained hunting dog and remove the olfactory bulbs, and then, after a sufficient period had elapsed to enable the animal to recover from the effects of the operation, to employ him again in field sports. If, after such an operation, the function still remains, there can be little question that it must be due to the impressions made upon the fifth pair.

Leaving this as a question to be determined by further investigation, and passing to the lingual branch of the fifth pair which is distributed to the anterior and lateral parts of the tongue, it may be stated, after much discussion of the question, which is the nerve of taste, the glosso-pharyngeal or the lingual branch of the fifth pair, physiologists have almost unanimously arrived at the conclusion that both nerves are concerned in this function; the first supplying the posterior third of the tongue and soft palate, and the second the anterior two-thirds of the tongue with the function of taste.

These conclusions have been adopted after numerous experiments, in which the loss of the sense of taste and of common sensation in the anterior part of the tongue followed immediately after the division of the fifth pair, or its lingual branch; while it still remained in the parts to which the glosso-pharyngeal nerve is distributed.

Of the branches of the fifth pair, which are distributed to the upper and lower teeth, five or six filaments are sent into every fang; these ascend and form a rich plexus in the upper part of the pulp by gradually breaking up into primitive fibres. According to Tomes, these fibres pass into the dentinal tubuli. Although this discovery is in accordance with a hypothesis long entertained, it has not, as yet, been thoroughly confirmed by other microscopists.*

It is of course well known, that the presence of the nerves in the tooth pulp endows it with that keen sensibility which characterizes that structure, and that the exquisite sensibility which is sometimes observed in dentine has been accounted for by the supposition that filaments of the nerve pass into the dentinal tubuli. In addition to this, in a former communication on the "Absorption of the Fangs of the Deciduous Teeth," the suggestion was advanced that the fifth pair of nerves had considerable influence over the nutrition of the dental tissues; and reasoning from the facts afforded by experiments, in which the nutrition of the special organs of sense had been affected by division of the fifth pair, it was inferred by analogy that the absorption of the fangs of the deciduous and the development of the permanent teeth were more or less dependent upon nervous connection.

(To be continued.)

